# Primary Osteosarcoma of the Bone with Rhabdoid Features: A Rare, Previously Undescribed Primary Malignant Tumor of Bone

**DOI:** 10.1155/2016/5901769

**Published:** 2016-12-12

**Authors:** Max Seiter, Motasem Al Maaieh, Andrew Rosenberg, Sheila Conway

**Affiliations:** ^1^University of Miami/Jackson Memorial Hospital Orthopaedic Surgery, Miami, FL, USA; ^2^University of Miami/Jackson Memorial Hospital Musculoskeletal Pathology, Miami, FL, USA

## Abstract

Primary osteosarcoma of the bone with rhabdoid features is a rare malignant tumor of bone, not previously described in the literature. Here we report a 69-year-old female who originally presented with a right femur pathologic fracture. Radiographs of the injury showed an aggressive-appearing lesion of the distal femur. Initial biopsy was done, which was not diagnostic; additional advanced imaging studies were performed, which failed to show any other site within the body with detectable disease process. Accordingly, the patient underwent radical resection of the distal femur and reconstruction with endoprosthesis. Histopathology obtained from the operative specimen showed osteosarcoma with rhabdoid features. Two months after surgery, the patient is symptom-free and doing well; she is currently pending adjuvant chemotherapy. Although rhabdoid features have been described in extraskeletal osteosarcoma, this appears to be the first mention of osteosarcoma of bone with rhabdoid features in the literature.

## 1. Introduction

**Figure 1:**
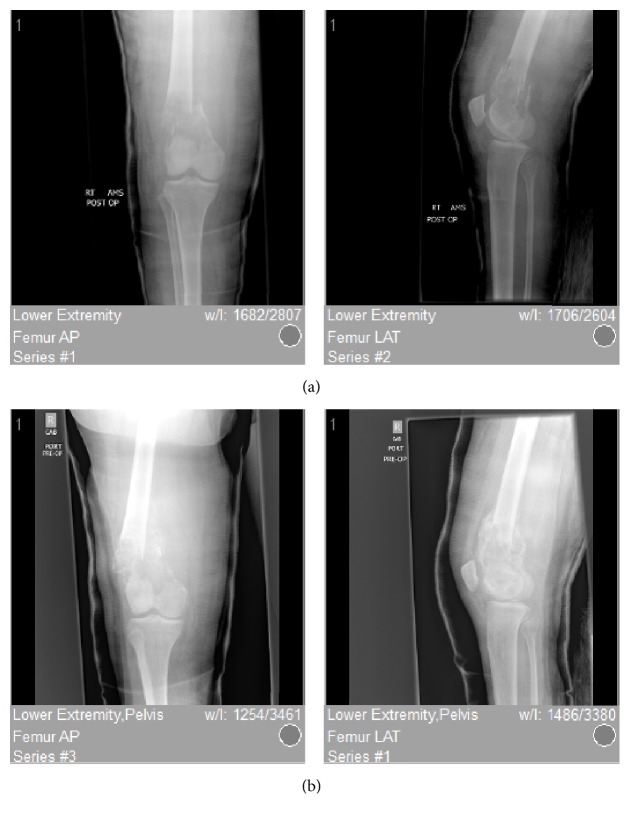
Preoperative AP/lateral radiographs showing an expansile, aggressive-appearing, lytic lesion of the distal femur with associated extra-articular pathologic fracture. (a) shows prereduction AP and lateral radiographs of a left distal femur pathologic fracture; (b) shows AP and lateral radiographs status, after reduction and splinting.

Primary osteosarcoma with rhabdoid features is a rare, previously undescribed malignant neoplasm of bone with histologic features of both primary osteosarcoma and those of rhabdoid cells. Osteosarcoma is mesenchymal neoplasm which accounts for 15% of all primary tumors of bone [1, 2]. Primary osteosarcoma is a highly aggressive tumor, with a bimodal distribution; its incidence peaks in the second decade of life and again spikes late in life, in older adults with Paget's disease [3]. Lesions are typically metaphyseal and most commonly seen at areas of high bone-turnover: the distal femur, proximal tibia, and proximal humerus [1, 4]. The World Health Organization has divided osteosarcoma into eight classifications based upon both anatomic location and histology: conventional, secondary, telangiectatic, small-cell, low-grade, parosteal, periosteal, and high-grade surface types [5]. Furthermore these may be subdivided according to predominant matrix produced: osteoblastic, chondroblastic, or fibroblastic [1, 4]. The defining histologic feature for the diagnosis of all osteosarcoma variants is the presence of mesenchymal cells producing osteoid [3]. These cells are spindle to polyhedral in shape, with pleomorphic nuclei and occasional mitotic figures [1]. Osteoid seen on histopathology has a distinct appearance: eosinophilic, dense, and amorphous [4].

Rhabdoid tumors have been divided into three classes, based upon anatomic location: malignant rhabdoid tumor of the kidney, extrarenal extracranial rhabdoid tumor, and atypical teratoid rhabdoid tumor involving the central nervous system [6]. Rhabdoid tumors were first identified in the kidney and were labeled as a rhabdomyosarcomatous variant of Wilms tumor [7]. Establishment of rhabdoid tumors as a distinct and separate entity from Wilms' variant occurred following the discovery of rhabdoid tumors in nearly every anatomical site. These rhabdoid tumors were recognized to feature similar characteristics, separate from the immunohistochemical and ultrastructural features of rhabdomyosarcoma [8]. When rhabdoid cells are seen in sarcomas, they signify an aggressive biological behavior of the tumor; and their presence portends a worse prognosis [9, 10]. Morphologically, rhabdoid cells are characterized by large polygonal cells, with large, eccentric, vesicular nuclei with prominent macronucleoli and copious eosinophilic cytoplasm, with classic juxtanuclear hyaline-like inclusions or globules [6, 11].

Extraskeletal osteosarcoma with rhabdoid features has been previously described twice in the literature, first in the scalp by Pillay et al. [12] and later in the temporal skin by Llamas-Velasco et al. [13]. However, although rhabdoid features have been observed in sarcomas and other extrarenal sites, this case report appears to be the first case of an osteosarcoma of bone with rhabdoid features.

## 2. Case History

**Figure 2:**
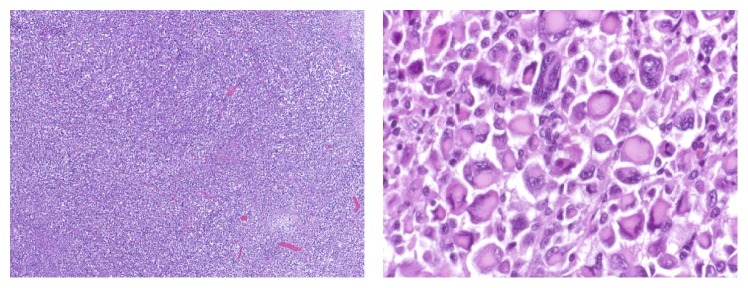
Low and high resolution slides from Temno needle biopsy of the distal femoral lesion showing rhabdoid-appearing cells without the presence of osteoid.

An otherwise healthy 69-year-old female presented to our emergency department with five days of acute right thigh pain and inability to ambulate after a low-energy fall from standing height. Prior to her acute presentation, the patient suffered from approximately 1 month of progressive knee and thigh pain; however she did not seek any medical care during that time period for her insidious lower extremity pain. The patient denied any constitutional symptoms, weight loss, or presence of other alarming symptoms during the time period leading up to her fracture. Initial radiographs of the right femur showed a pathologic fracture associated with an aggressive-appearing lesion in the distal femur. The associated lesion was a markedly expansile, poorly geographically defined lytic lesion measuring approximately 9 × 6.7 cm in the metaphysis of the distal femur. The films also revealed associated periosteal reaction with disorganized bone formation and cortical destruction laterally; the pathological fracture could be observed at the proximal extent of the lesion (Figure 1(a)). Due to these findings and the concern for malignancy, the patient underwent a standard workup including CT chest/abdomen/pelvis and PET scan; no primary lesions or metastases were found. With no clear diagnosis and no evidence of lesions elsewhere in the body, the decision was made to obtain a tissue diagnosis in order to further guide the patient's treatment. Our patient accordingly was taken to the operating room for a fluoroscopically guided needle biopsy and closed reduction and casting under anesthesia (Figure 1(b)). The sample obtained from the needle biopsy featured rhabdoid cells when viewed under the microscope (Figure 2). Importantly, there was no osteoid noted in this original sample.

**Figure 3:**
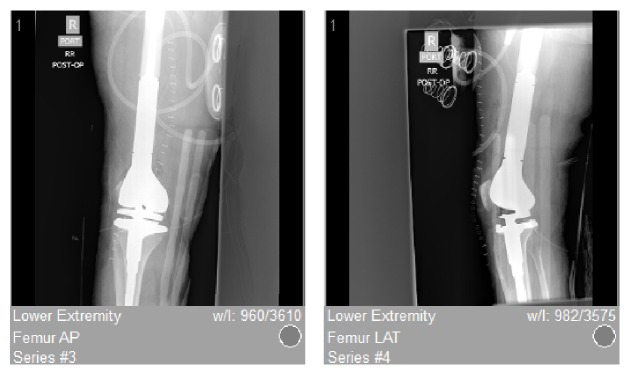
Figure 3 shows postoperative AP/lateral radiographs status, after wide resection of the lesion and replacement with distal femoral endoprosthesis.

Given the lack of osteoid-producing cells seen on original histopathology, the differential diagnosis at that time did not feature osteosarcoma. Taking into account the presence of rhabdoid cells, the working diagnosis at that time was established as metastatic carcinoma of unknown origin with rhabdoid features, versus rhabdoid sarcoma of the thigh with local spread to the femur. Therefore, the patient was referred for chemotherapy; however, she experienced issues with payment due to the expiration of her insurance, and she was unable to initiate chemotherapeutic treatment. The patient's leg remained in a long leg cast, and she was lost to follow-up for a brief period of time. One month later, she again presented to the emergency department, now with severe pain, inability to ambulate, balance problems, and high fall risk. Due to the patient's unclear diagnosis, her inability to obtain chemotherapy, and the few reasonable treatment options in this setting, the decision was made to treat patient with right distal femur radical resection and reconstruction with an endoprosthesis (Figure 3). The procedure was done uneventfully, and there were no complications.

**Figure 4:**
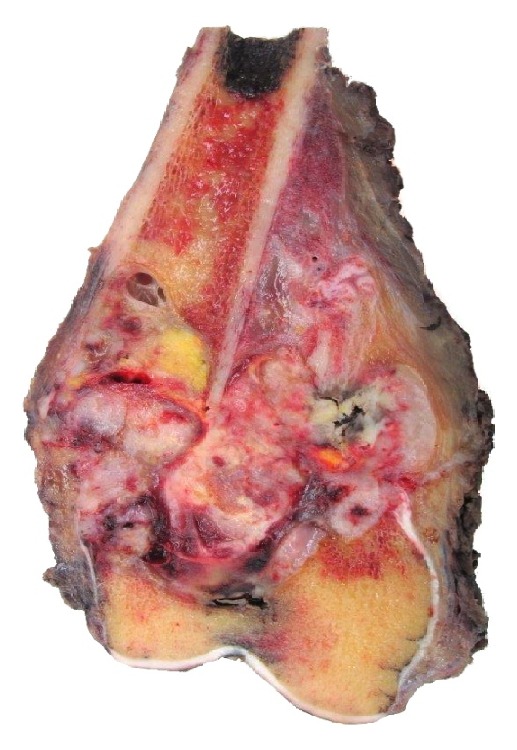
Showing gross pathology of the resected tumor showing an aggressive lesion with expansion beyond the cortices of the distal femur and into the soft tissue and new bone formation within the lesion.

**Figure 5:**
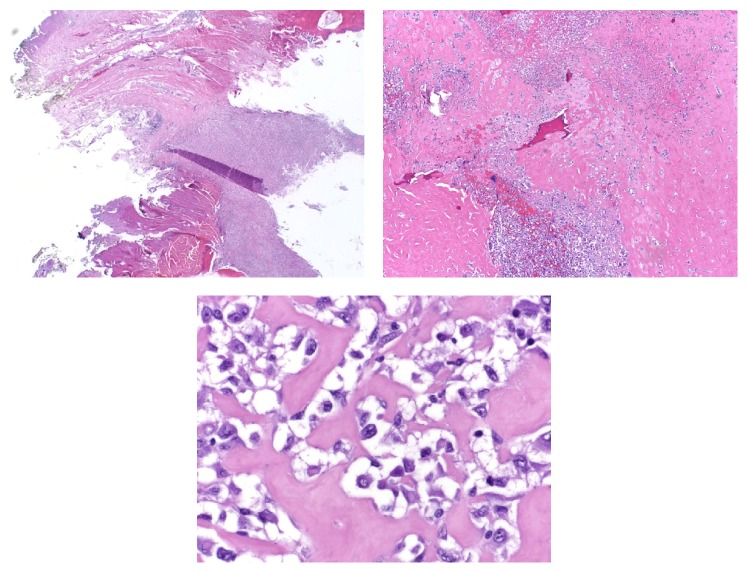
Showing low, intermediate, and high resolution histopathology slide prominently featuring rhabdoid-appearing cells producing osteoid which is pathognomonic for osteosarcoma.

Gross pathology of the surgical specimen showed the presence of new, disorganized bone formation (Figure 4); and histopathology of the specimen featured osteoid-producing cells and osteoid, in the presence of numerous amounts of rhabdoid-appearing cells (Figure 5). With the discovery of osteoid and osteoid-producing cells in the pathologic specimen, which had been previously absent from the sample obtained by needle biopsy, the diagnosis was revised to osteosarcoma with prominent rhabdoid cells, high grade 3/3, 9 cm, with pathologic fracture and callous formation. Immunohistochemical staining with special AT-rich sequence-binding protein 2 (SATB2) was positive. The procedure went without complications, the patient tolerated the procedure well, and she had an uneventful postoperative course. The patient was also referred for adjuvant chemotherapy, following her radical resection, as it is standard of care; but due to the barriers that previously prevented her access to care, she has unfortunately yet to seek out treatment.

## 3. Discussion

The most common malignancy of bone is metastatic, particularly in the elderly population, which would have made a metastatic lesion a likely probability in our patient. However a thorough workup via CT and PET imaging showed no other lesions in the patient's body. Furthermore the imaging revealed multiple characteristics of osteosarcoma, such as a sunburst pattern of periosteal reaction, new disorganized bone formation extending into adjacent tissue, cortical destruction, and the location of the lesion. Nonetheless, malignancy of unknown primary source was still felt to be the most likely diagnosis after the patient's needle biopsy, given the findings of rhabdoid cells and lack of any finding of osteoid matrix (Figure 2).

The working diagnosis was revised however, after histopathology obtained from the surgical specimen showed the presence of osteoid matrix and matrix-producing cells. As in the original needle biopsy sample, eosinophilic rhabdoid cells with their characteristic nuclei and nucleoli and cytoplasmic inclusions were prominently featured and clearly identified under the microscope (Figures 2 and 5). However the ubiquitous presence of osteoid in association with these cells was distinctly evident in the surgical specimen (Figure 5) and accordingly the diagnosis was revised to primary osteosarcoma of the bone with rhabdoid features. Further, validating this diagnosis, staining with special AT-rich sequence-binding protein 2 (SATB2) was positive. SATB2 is a nuclear matrix protein that plays a critical role in osteoblast lineage commitment [14]. It is a sensitive and specific immunohistochemical marker of osteoblastic differentiation in bone and soft tissue tumors and is seen in nearly all cases of osteosarcoma [15]. It has been recognized to be of high utility in cases in which histological features of matrix are equivocal or in which biopsy only samples undifferentiated cells. Importantly, soft tissue tumors, such as sclerosing rhabdomyosarcoma, with abundant hyalinized collagen are categorically negative for SATB2 [15]. Accordingly, the presence of SATB2 in the rhabdoid-appearing cells in our specimen helps to confirm the diagnosis of an osteosarcoma with prominent rhabdoid features. The presence of ubiquitous rhabdoid cells and an osteosarcomatous appearance on pathology in conjunction with SATB2 positivity is why we have identified this a unique entity. Were the rhabdoid cells not present, then a diagnosis such as conventional osteoblastic medullary osteosarcoma would be appropriate, but it is the presence of the rhabdoid cells that leads us to the novel diagnosis.

The question remains however: is this a primary osteosarcoma of bone? The incidence of osteosarcoma is bimodal; the second, smaller peak appears in adults greater than 65 years of age [16]. Our patient was aged 69 and was within this age range, but her case differed from that of the standard patient in this demographic. The second peak of osteosarcoma incidence is generally attributed to secondary malignancies [16], such as those secondary to Paget's disease or other processes known to elevate the risk of developing osteosarcoma. Paget's disease is a diagnosis our patient did not carry; furthermore her preoperative imaging showed no evidence of any similar processes, nor did she have any known risk factors such as a history of prior irradiation. In the absence of these factors, without other known lesions, and with the aforementioned histopathology which features prominent osteoid production, the patient's diagnosis is most correctly defined as a primary osteosarcoma of bone.

This patient's diagnosis, primary osteosarcoma of bone with rhabdoid features, is an apparently novel diagnosis, not yet described in the literature. Unlike the bimodal distribution observed in osteosarcoma, rhabdoid tumors are almost exclusively pediatric tumors. Typically, rhabdoid tumors are found in the central nervous system or in the kidneys [11]. There have been reported cases of local invasion of rhabdoid tumors from the spinal cord to bone [17], as well as metastases from kidney to bone [18]. However, these are examples of local and distant metastatic invasion, not primary tumor of bone. There are also cases of extrarenal extracranial rhabdoid tumor (EERT) with diffuse presentations involving bone at birth [19], as well as case reports of bone metastases in older adults from EERT locations such as bladder [20], pancreas [21], and colon [22], again, though these are examples of local or distant metastatic invasion, not primary tumors of bone origin. With the exception of our above described case, there has been no description of primary osteosarcoma of bone with rhabdoid features in the literature.

## References

[1] Messerschmitt P. J., Garcia R. M., Abdul-Karim F. W., Greenfield E. M., Getty P. J. (2009). Osteosarcoma. *Journal of the American Academy of Orthopaedic Surgeons*.

[2] Murphey M. D., Robbin M. R., McRae G. A., Flemming D. J., Temple H. T., Kransdorf M. J. (1997). The many faces of osteosarcoma. *Radiographics*.

[3] Gorlick R. G., Toretsky J. A., Marina N., Kufe D. W., Pollock R. E., Weichselbaum R. R. (2003). Osteosarcoma. *Holland-Frei Cancer Medicine*.

[4] Green J. T., Mills A. M. (2014). Osteogenic tumors of bone. *Seminars in Diagnostic Pathology*.

[5] Fletcher C. M., Unni K. K., Mertens F. (2002). Pathology & genetics of tumors of soft tissue and bone. *World Health Organization Classifications of Tumors*.

[6] Uwineza A., Gill H., Buckley P. (2014). Rhabdoid tumor: the Irish experience 1986–2013. *Cancer Genetics*.

[7] Beckwith J. B., Palmer N. F. (1978). Histopathology and prognosis of Wilms tumor. results from the first national Wilms' tumor study. *Cancer*.

[8] Haas J. E., Palmer N. F., Weinberg A. G., Beckwith J. B. (1981). Ultrastructure of malignant rhabdoid tumor of the kidney. A distinctive renal tumor of children. *Human Pathology*.

[9] Wick M. R., Ritter J. H., Dehner L. P. (1995). Malignant rhabdoid tumors: a clinicopathologic review and conceptual discussion. *Seminars in Diagnostic Pathology*.

[10] Oda Y., Tsuneyoshi M. (2006). Extrarenal rhabdoid tumors of soft tissue: clinicopathological and molecular genetic review and distinction from other soft-tissue sarcomas with rhabdoid features. *Pathology International*.

[11] Alaggio R., Boldrini R., Venosa B. D., Rosolen A., Bisogno G., Magro G. (2009). Pediatric extra-renal rhabdoid tumors with unusual morphology: a diagnostic pitfall for small biopsies. *Pathology Research and Practice*.

[12] Pillay P., Simango S., Govender D. (2000). Extraskeletal osteosarcoma of the scalp. *Pathology*.

[13] Llamas-Velasco M., Rütten A., Requena L., Mentzel T. (2013). Primary cutaneous osteosarcoma of the skin: a report of 2 cases with emphasis on the differential diagnoses. *American Journal of Dermatopathology*.

[14] Dobreva G., Chahrour M., Dautzenberg M. (2006). SATB2 Is a Multifunctional Determinant of Craniofacial Patterning and Osteoblast Differentiation. *Cell*.

[15] Conner J. R., Hornick J. L. (2013). SATB2 is a novel marker of osteoblastic differentiation in bone and soft tissue tumours. *Histopathology*.

[16] Ottaviani G., Jaffe N. (2009). The epidemiology of osteosarcoma. *Cancer Treatment and Research*.

[17] Warmuth-Metz M., Bison B., Gerber N. U., Pietsch T., Hasselblatt M., Frühwald M. C. (2013). Bone involvement in atypical teratoid/rhabdoid tumors of the CNS. *American Journal of Neuroradiology*.

[18] Park S., Seo J.-H., Park J. B., Park S. (2014). Malignant rhabdoid tumor of the kidney and spine in an infant. *Journal of Korean Neurosurgical Society*.

[19] Sajedi M., Wolff J. E. A., Egeler R. M. (2002). Congenital extrarenal non-central nervous system malignant rhabdoid tumor. *Journal of Pediatric Hematology/Oncology*.

[20] Pasricha S., Hafiz A., Gandhi J. S., Mehta A. (2011). Urothelial carcinoma of bladder having rhabdoid differentiation with isolated scapular metastasis. *Journal of Cancer Research and Therapeutics*.

[21] Sironi M., Grando D., Spinelli M. (2001). Bone marrow metastatic infiltration of a rabdoid pancreatic tumor. *Haematologica*.

[22] Cho I.-J., Kim S.-S., Min Y.-D., Noh M.-W., Hong R. (2015). Poorly differentiated cecal adenocarcinoma showing prominent rhabdoid feature combined with appendiceal mucinous cystadenoma: a case report and review of the literature. *Oncology Letters*.

